# Editorial: Microsatellite and microsatellite instability

**DOI:** 10.3389/fgene.2023.1189212

**Published:** 2023-05-30

**Authors:** Alexandre How-Kit, Kai Ye

**Affiliations:** ^1^Laboratory for Genomics, Foundation Jean Dausset—CEPH, Paris, France; ^2^MOE Key Lab for Intelligent Networks and Networks Security, Faculty of Electronic and Information Engineering, Xi’an Jiaotong University, Xi’an, China; ^3^ School of Automation Science and Engineering, Faculty of Electronic and Information Engineering, Xi’an Jiaotong University, Xi’an, China; ^4^ Genome Institute, The First Affiliated Hospital of Xi’an Jiaotong University, Xi’an, China; ^5^ The School of Life Science and Technology, Xi’an Jiaotong University, Xi’an, China; ^6^Faculty of Science, Leiden University, Leiden, Netherlands

**Keywords:** microsatellite instability, polymerase slippage, cancer, immunotherapy, HSP110 HT17, biomarker, next-generation sequencing – NGS

Microsatellite instability (MSI) is a genome-wide genetic alteration initially discovered in colorectal cancers (CRC) in 1993, where about 15% of tumors presented this phenotype that bears relevant information for diagnostics, prognostics and prediction of therapeutic response (Boland and Goel, 2010). MSI is characterized by the accumulation of mutations–insertions or deletions of a few nucleotides–in microsatellites, introduced during replication by polymerase slippage (Boland and Goel, 2010). MSI is caused by the loss of function of one of the DNA mismatch repair system (MMR) proteins (mostly in MLH1, MSH2, PMS2 and MSH6) due to somatic and/or inherited inactivating (epi)mutations of their gene (Boland and Goel, 2010). With the advent of next-generation sequencing (NGS) in the era of genomics, several pan-cancer investigations on publicly available whole genome/exome sequencing (WGS/WES) cancer data highlighted the presence of MSI in most solid cancer types at variable frequencies (<1%–∼30%) with tumor-specific MSI signatures (Hause et al., 2016; Cortes-Ciriano et al., 2017). Concomitantly, the development and availability of immune checkpoint blockade therapies (ICI) for cancer patients allowed the identification of MSI as a major predictor of treatment response, first in CRC (Le et al., 2015) and soon after in all solid cancers (Le et al., 2017). This led the FDA to approve the use of MSI status as a universal biomarker for the administration of immune checkpoint inhibitors in advanced solid cancers, regardless of the type of cancers.

Due to the considerable clinical implication of MSI for cancer patient’s management, the study of MSI in cancer was marked by a renewed interest in the biomedical community, either for basic researches, development of new approaches for MSI detection, or assessment of its clinical significance in various cancer types and sub-types. In this Research Topic dedicated to ‘Microsallite and Microsatellite Instability’, Aska et al., presented a fundamental study of genome-wide single-cell mononucleotide microsatellites of MMR-proficient and MMR-deficient thymic T cells in mouse model, aiming to decipher the mutationnal dynamics of MSI *in vivo*. By comparing WES data from *Mlh1* ^*−/−*^ and *Mlh1* ^*+/+*^ T cells, their analyses revealed several specific genomic features of MSI in MMRD cells, distinguishing deletion and insertion dynamics (Aska et al.). They notably showed that deletions far outnumbered insertions as in MSI tumors and also preferentially affected long A/T mononucelotide microsatellites of 10–14 nt and later-replicating genes (Aska et al.). Moreover, their results indicated that longer-than-average and transcriptionnally active genes were areas of fragility more prone to MSI, which could potentially explain why some microsatellites are more unstable than others depending on the cancer type.

In the second article of this Research Topic, Han et al., proposed an overall and updated review that summerized the clinical, genomic and tumor immunobiology of sporadic and familial MSI cancers with a particular attention to metastatic or recurrent disease. They notably focused in three cancer types with high frequencies of MSI, i.e., colorectal, gastric and endometrial cancer and emphatised in the differences of MSI cancers compared to their microsatellite stable (MSS) counterparts (Han et al.). Treatment options and results of clinical trials were discussed, including notably patients response to ICI and resistance mechansims, and they suggested several perspectives about the possible evolution of MSI cancer treaments including multi-combined therapies.

In a third study, Liu et al., indentifyed a fatty-acid metabolism-related (FAM) gene signature from colon adenocarcinoma TCGA RNA-seq data and developed a FAM risk-score associated with a better prognostic value (AUC = 0.734) than other clinicopathological parameters. In addition, they showed that the high-risk group of CRC patients was enriched in MSI (high and low) phenotypes compared to the low-risk group and sugested that their risk score might also be a predicator of chemotherapy or immunotherapy (Liu et al.). Although further validations are needed for this FAM risk score, identifying new predicators of ICI is still highly valuable as neither MSI, TMB nor PD-L1 expression alone can fully predict response to ICI and they should be combined with other biomarkers to improve prediction performances in the different cancer types and sub-types.

The gold-standard approach for MSI detection in cancer still remains to date PCR and capillary electrophoresis fragment analysis of a panel of five microsatellites, which can be either the pentaplex or the first recommended NCI/Bethesda panel. The NCI/Bethesda panel was proposed for MSI CRC and Lynch syndrome diagnosis but presented several limitations and Research Topic, notably due to use of three highly polymorphic di-nucleotide microsatellites (Umar et al., 2004; Baudrin et al., 2018). Several groups proposed alternative markers for improved MSI detection, including HT17, a quasi-monomorphic mononucleotide microsatellite located in the *HSP110* gene that showed improved sensitivity for similar specificity in CRC compared to the pentaplex panel (Buhard et al., 2016), while the size of HT17 deletions could also be a predictor of 5-FU–based chemotherapy response in CRC patients (Collura et al., 2014). Tachon et al., performed a comprehensive evaluation of HT17 as a diagnostic and prognostic biomarker in a cohort of 381 MSI CRC patients, including 280 stage II and III patients of whom 37% received adjuvant chemotherapy mostly based (80.6%) on 5-FU associated with oxaliplatin. Interestingly, their results confirmed HT17 as a good marker for MSI detection achieving 95.5% sensitivity, even allowing the detection of false-positive MSI samples by the pentaplex panel. However, neither HSP110 expression nor deletion size correlated with time to recurrence in patients with stage II and III CRC having received or not an adjuvant chemotherapy (Tachon et al.), highlighting the importance of validation studies on independent cohorts.

In conclusion, this Research Topic on ‘Microsatellite and Microsatellite Instability’ covers different aspects of basic, translational and clinical research on MSI, including its importance in oncology for cancer patients’ management. We thank the contributing authors and hope that their papers will be appreciated by their readers and that the interest for microsatellites and MSI will continue to grow within the biomedical community.
